# Supplementary material for: Efficacy and safety of low molecular weight heparin compared to unfractionated heparin for chronic outpatient hemodialysis in end stage renal disease: systematic review and meta-analysis
Source: PeerJ. 2015 Mar 10;3:e835. doi: 10.7717/peerj.835 (PMC4359121; doi:10.7717/peerj.835)
Supplement: Supplemental Information 1 — Search strategy for Medline, Embase and Cochrane library. [file peerj-03-835-s001.docx]

**Search strategy:** Our final combined Pubmed Search stragety will be as follows:
1) ("randomized controlled trials as topic"[MeSH Terms] OR ((randomized controlled trial[pt] OR controlled clinical trial[pt] OR randomized[tiab] OR placebo[tiab] OR "drug therapy"[Subheading] OR randomly[tiab] OR trial[tiab] OR groups[tiab]) NOT ("animals"[MeSH Terms] NOT "humans"[MeSH Terms]))) - Highly sensitive cochrane search strategy for RCTs AND

2) ("Anticoagulants"[Mesh] OR "Anti-coagulants"[All Fields] OR "Anti-coagulant"[All Fields] OR “anticoagulant”[all fields] OR “antithrombin”[all fields] OR "Anticoagulant Agents"[All Fields] OR "Anticoagulant Agents"[All Fields] OR "Anticoagulant Drugs"[All Fields] OR "Antithrombins"[Mesh] OR "Antithrombins"[All Fields] OR "Heparin, Low Molecular Weight"[MESH] OR “Adomiparin”[all fields] OR “antixarin”[all fields] OR “ardeparin”[all fields] OR “bemiparin”[all fields] OR “certoparin”[all fields] OR “cy 222”[all fields] OR “danaparoid”[all fields] OR “deligoparin”[all fields] OR “embolex”[all fields] OR “idrabiotaparinux”[all fields] OR “idraparinux”[all fields] OR “livaparin”[all fields] OR “minolteparin”[all fields] OR “monoembolex”[all fields] OR “parneparin”[all fields] OR “rd 11885”[all fields] OR “reviparin”[all fields] OR “semuloparin”[all fields] OR “tafoxiparin”[all fields] OR “tedelparin”[all fields] OR “logiparin”[all fields] OR “eurodal”[all fields] OR “boxol”[all fields] OR “low liquemine”[all fields] OR “enoparin”[all fields] OR “decipar”[all fields] OR “henoxil”[all fields] OR “hepaclex”[all fields] OR “lomoh”[all fields] OR “nu-mox”[all fields] OR “plaucina”[all fields] OR "Dalteparin"[MESH] OR "Enoxaparin"[MESH] OR "Heparin, Low Molecular Weight"[all fields] OR "LMWH"[all fields] OR "Low Molecular Weight Heparin"[all fields] OR "Low-Molecular Weight Heparin"[all fields] OR "Low-Molecular-Weight Heparin"[all fields] OR "Low Molecular-Weight Heparin"[all fields] OR "LMW Heparin"[all fields] OR "LMW-Heparin"[all fields] OR "Tinzaparin"[all fields] OR "Innohep"[all fields] OR "tinzaparin sodium"[All Fields] OR "innohep"[All Fields] OR "logiparin"[All Fields] OR "Fondaparinux"[all fields] OR "Arixtra"[all fields] OR "Dalteparin"[all fields] OR "Fragmin"[all fields] OR "Dalteparin Sodium"[all fields] OR "Tedelparin"[all fields] OR "Kabi-2165"[all fields] OR “kabi2165”[all fields] OR “kabi 2165”[all fields] OR "Kabi 2165"[all fields] OR "Fragmin"[all fields] OR "Fragmine"[all fields] OR "Dalteparin Sodium"[all fields] OR "Sodium, Dalteparin"[all fields] OR "FR-860"[all fields] OR "FR 860"[all fields] OR “FR860”[all fields] OR "Enoxaparin"[all fields] OR "Lovenox"[all fields] OR "Enoxaparin"[all fields] OR "Enoxaparine"[all fields] OR "PK-10,169"[all fields] OR "PK 10,169"[all fields] OR "PK-10169"[all fields] OR "PK 10169"[all fields] OR "PK10169"[all fields] OR "EMT-967"[all fields] OR "EMT 967"[all fields] OR "EMT-966"[all fields] OR "EMT 966"[all fields] OR “Clexan”[all fields] OR "Clexane"[all fields] OR "Cutenox"[all fields] OR "Dripanina"[all fields] OR "Enoxaparin Sodium"[all fields] OR "Klexane"[all fields]): Search Strategy for anti-coagulants and heparin.

AND

3) ("Renal Dialyses"[all fields] OR "Renal Dialysis"[MeSH] OR "hemodialysis"[all fields] OR "Hemodialyses"[all fields] OR "Extracorporeal Dialyses"[all fields] OR "Haemodialysis"[all fields] OR "Dialysis"[MeSH] OR "Dialysis"[all fields] OR "Microdialysis"[MeSH] OR "Microdialysis"[all fields] OR "Hemodiafiltration"[mesh] OR "hemodiafiltration"[all fields] OR "hemo-dialysis"[all fields] OR "hemo-dialyses"[all fields] OR "haemodialyses"[All Fields] OR “renal replacement therapy”[all fields] OR "RRT"[all fields]): Search strategy for dialysis.

Final Search: #1 AND #2 AND # 3

**Cochrane CENTRAL Search**: Our Cochrane search strategy will be as follows:

#1 MeSH descriptor Anticoagulants explode all trees     
#2 MeSH descriptor Antithrombins explode all trees
#3 MeSH descriptor Heparin, Low-Molecular-Weight explode all trees
#4 (anticoagulant agent) OR heparin OR (low molecular weight heparin) OR Adomiparin OR (antixarin) OR (ardeparin) OR (bemiparin) OR (certoparin) OR (cy 222) OR (dalteparin) OR (danaparoid) OR (deligoparin) OR (embolex) OR (enoxaparin) OR (fondaparinux) OR (idrabiotaparinux) OR (idraparinux) OR (livaraparin calcium) OR (minolteparin) OR (monoembolex) OR (nadroparin) OR (parnaparin) OR (rd 11885) OR (reviparin) OR (semuloparin) OR (tafoxiparin) OR (tedelparin) OR (tinzaparin) OR (heparin derivative)/exp OR (anticoagulant agent)/exp OR (Anticoagulants) OR (Anti-coagulants) OR (Anti-coagulant) OR (Anticoagulant) OR (Antithrombins) OR (Low Molecular Weight Heparin) OR (Low Molecular Weight Heparins) OR (LMWH) OR (Low-Molecular Weight Heparin) OR (Low-Molecular Weight Heparins) OR (Low Molecular-Weight Heparin) OR (Low Molecular-Weight Heparins) OR (LMW Heparin) OR (LMW-Heparin) OR (tinzaparin) OR (innohep) OR (logiparin) OR (Arixtra) OR (Fragmin) OR (Tedelparin) OR (Kabi-2165) OR (Kabi 2165) OR (Kabi2165) OR (Fragmine) OR (FR-860) OR (FR 860) OR (FR860) OR (Eurodal) OR (Boxol) OR (Low Liquemine) OR (Lovenox ) OR (Enoxaparine) OR (PK-10,169)  OR (PK 10,169) OR (PK10,169) OR (PK-10169) OR (PK 10169) OR (PK10169) OR (EMT-967) OR (EMT 967) OR (EMT967) OR (EMT-966) OR (EMT 966) OR (EMT966) OR (Clexa) OR (Clexane) OR (Cutenox) OR (Decipar) OR (Dripanina) OR (Enoparin) OR (Flenox) OR (Henoxil) OR (Hepaclex) OR (Klexane) OR (Lomoh) OR (Nu-Rox) OR (Plaucina) OR (Trombenox)
#5 MeSH descriptor Dialysis explode all trees
#6 MeSH descriptor Hemodiafiltration explode all trees.

#7 MeSH descriptor Hemodialysis, Home explode all trees
#8 (Renal Dialyses) OR Hemodialysis OR Hemodialyses OR (Extracorporeal Dialyses) OR Dialysis OR Microdialysis OR Hemodiafiltration OR hemodiafiltration OR haemodialyses OR dialyses OR haemodialysis OR (renal replacement therapy)
#9 ((#1 OR #2 OR #3 OR #4) AND (#5 OR #6 OR #7 OR #8)): will be the final combined search strategy for Cochrane central.

**EMBASE Search**:
#1 Anti-coagulant part -
‘anticoagulant agent’/exp OR ‘heparin’/exp OR ‘low molecular weight heparin’/exp OR ‘Adomiparin’ OR ‘antixarin’ OR ‘ardeparin’ OR ‘bemiparin’ OR ‘certoparin’ OR ‘cy 222’ OR ‘dalteparin’ OR ‘danaparoid’ OR ‘deligoparin’ OR ‘embolex’ OR ‘enoxaparin’ OR ‘fondaparinux’ OR ‘idrabiotaparinux’ OR ‘idraparinux’ OR ‘livaraparin calcium’ OR ‘minolteparin’ OR ‘monoembolex’ OR ‘nadroparin’ OR ‘parnaparin’ OR ‘rd 11885’ OR ‘reviparin’ OR ‘semuloparin’ OR ‘tafoxiparin’ OR ‘tedelparin’ OR ‘tinzaparin’ OR ‘heparin derivative’/exp OR ‘anticoagulant agent’/exp OR “Anticoagulants” OR “Anti-coagulants” OR “Anti-coagulant” OR “Anticoagulant” OR “Antithrombins” OR “Low Molecular Weight Heparin” OR “Low Molecular Weight Heparins” OR “LMWH” OR “Low-Molecular Weight Heparin” OR “Low-Molecular Weight Heparins” OR “Low Molecular-Weight Heparin” OR “Low Molecular-Weight Heparins” OR “LMW Heparin” OR “LMW-Heparin” OR “tinzaparin” OR “innohep” OR “logiparin” OR “Arixtra” OR “Fragmin” OR “Tedelparin” OR “Kabi-2165” OR “Kabi 2165” OR “Kabi2165” OR “Fragmine” OR “FR-860” OR “FR 860” OR “FR860” OR “Eurodal” OR “Boxol” OR “Low Liquemine” OR “Lovenox ” OR “Enoxaparine” OR “PK-10,169”  OR “PK 10,169” OR “PK10,169” OR “PK-10169” OR “PK 10169” OR “PK10169” OR “EMT-967” OR “EMT 967” OR “EMT967” OR “EMT-966” OR “EMT 966” OR “EMT966” OR “Clexa” OR “Clexane” OR “Cutenox” OR “Decipar” OR “Dripanina” OR “Enoparin” OR “Flenox” OR “Henoxil” OR “Hepaclex” OR “Klexane” OR “Lomoh” OR “Nu-Rox” OR “Plaucina” OR “Trombenox”
#2 Dialysis part -
‘dialysis’/exp OR dialysis OR ‘hemodialysis’/exp OR hemodialysis OR ‘haemodialysis’/exp OR haemodialysis OR ‘extended daily dialysis’/exp OR ‘home dialysis’/exp OR ‘renal replacement therapy’/exp OR ‘renal replacement therapies’/exp OR ‘hemofiltration’/exp OR hemofiltration or ‘hemodiafiltration’/exp OR hemodiafiltration OR ‘hemo-dia-filtration’/exp OR hemo-dia-filtration OR ‘haemofiltration’/exp OR haemofiltration OR haemodiafiltration OR ‘haemodiafiltration’/exp OR ‘intermittent dialysis’/exp OR ‘outpatient dialysis’/exp OR ‘out-patient dialysis’/exp OR ‘outpatient hemodialysis’/exp OR ‘out-patient hemodialysis’/exp OR ‘outpatient haemodialysis’/exp OR ‘out-patient haemodialysis’/exp OR ‘outpatient hemofiltration’/exp OR ‘out-patient hemofiltration’/exp OR ‘out-patient hemodiafiltration’/exp OR ‘outpatient hemodiafiltration’/exp OR ‘renal dialysis’/exp OR ‘extracorporeal dialysis’/exp OR ‘extra-corporeal dialysis’/exp OR microdialysis OR ‘microdialysis’/exp OR micro-dialysis OR ‘micro-dialysis’/exp
#3 RCT part -
We combined (using OR) the ideas of the most sensitive and middle sensitive approaches from the Wong paper on EMBASE searching strategies for RCTs
random*:de,lnk,ab,ti,au OR random:de,lnk,ab,ti,au OR 'clinical trial':de,lnk,ab,ti,au OR 'clinical trials':de,lnk,ab,ti,au OR 'health care quality'/exp OR 'health care quality' OR 'treatment outcome'/exp OR ‘treatment outcome'
FINAL COMBO  #1 and #2 and #3 = final Embase search.
