# Supplementary material for: Efficacy and safety of low molecular weight heparin compared to unfractionated heparin for chronic outpatient hemodialysis in end stage renal disease: systematic review and meta-analysis
Source: PeerJ. 2015 Mar 10;3:e835. doi: 10.7717/peerj.835 (PMC4359121; doi:10.7717/peerj.835)
Supplement: Table S1 — Comparison of co-morbid conditions between the 2 groups. [file peerj-03-835-s003.docx]

| Comorbidities  (%) | Aggarwal | | Borm | | Elisaf | | Gritters | | Harenberg | | Hottelart | | Lane | |
| --- | --- | --- | --- | --- | --- | --- | --- | --- | --- | --- | --- | --- | --- | --- |
|  | LMWH | UH | LMWH | UH | LMWH | UH | LMWH | UH | LMWH | UH | LMWH | UH | LMWH | UH |
| Diabetes | 34 | 31 | 36 | 33 | 27 | 31 | 37 | 35 | 21 | 24 | 36 | 32 | 35 | 34 |
| Hypertension | 26 | 25 | 29 | 24 | 37 | 35 | 28 | 34 | 30 | 33 | 28 | 30 | 31 | 27 |
| CAD | 16 | 18 | NR | NR | NR | NR | 14 | 13 | NR | NR | NR | NR | 18 | 17 |
| Smoking | 21 | 23 | NR | NR | 18 | 19 | NR | NR | 17 | 21 | 24 | 25 | NR | NR |
| Obesity | 21 | 19 | NR | NR | NR | NR | NR | NR | NR | NR | 22 | 24 | NR | NR |
| Dyslipidemia | 27 | 30 | NR | NR | NR | NR | NR | NR | NR | NR | NR | NR | 23 | 22 |
| Comorbidities  (%) | Lord | | Verzan | | Naumnik 2009 | | Mahmood | | Naumnik 2003 | | Naumnik 2007 | | Naumnik 2007 | |
|  | LMWH | UH | LMWH | UH | LMWH | UH | LMWH | UH | LMWH | UH | LMWH | UH | LMWH | UH |
| Diabetes | 31 | 28 | 22 | 24 | 28 | 26 | 32 | 28 | 30 | 26 | 33 | 29 | 28 | 31 |
| Hypertension | 26 | 25 | 31 | 34 | 33 | 31 | 24 | 21 | 25 | 27 | 28 | 23 | 30 | 29 |
| CAD | 21 | 23 | NR | NR | NR | NR | NR | NR | NR | NR | NR | NR | 16 | 12 |
| Smoking | 17 | 19 | 21 | 18 | NR | NR | NR | NR | NR | NR | NR | NR | NR | NR |
| Obesity | NR | NR | NR | NR | NR | NR | 18 | 21 | 23 | 21 | 16 | 19 | 15 | 14 |
| Dyslipidemia | NR | NR | NR | NR | NR | NR | NR | NR | NR | NR | 21 | 18 | 20 | 21 |
| Comorbidities  (%) | Naumnik 2009 | | Poyrazoglu | | Ryan | | Saltissi | | Schrader | |  |  |  |  |
|  | LMWH | UH | LMWH | UH | LMWH | UH | LMWH | UH | LMWH | UH |  |  |  |  |
| Diabetes | 32 | 34 | 28 | 24 | 33 | 34 | 27 | 25 | 25 | 26 |  |  |  |  |
| Hypertension | 21 | 22 | 31 | 29 | 30 | 31 | ^*^28 | 21 | 24 | 29* |  |  |  |  |
| CAD | 15 | 17 | NR | NR | NR | NR | 15 | *22 | 14 | 15 |  |  |  |  |
| Smoking | 21 | 20 | NR | NR | NR | NR | 19 | 18 | 14 | 17 |  |  |  |  |
| Obesity | *24 | 18 | 22 | 17 | NR | NR | NR | NR | 21 | 20 |  |  |  |  |
| Dyslipidemia | NR | NR | NR | NR | NR | NR | NR | NR | NR | NR |  |  |  |  |

**Supplemental Table 1: Comorbid conditions of the study participants in the included studies**

NR: not reported,

*: indicates significant difference
