# Supplementary figures and images for: Efficacy and safety of low molecular weight heparin compared to unfractionated heparin for chronic outpatient hemodialysis in end stage renal disease: systematic review and meta-analysis
Source: PeerJ. 2015 Mar 10;3:e835. doi: 10.7717/peerj.835 (PMC4359121; doi:10.7717/peerj.835)

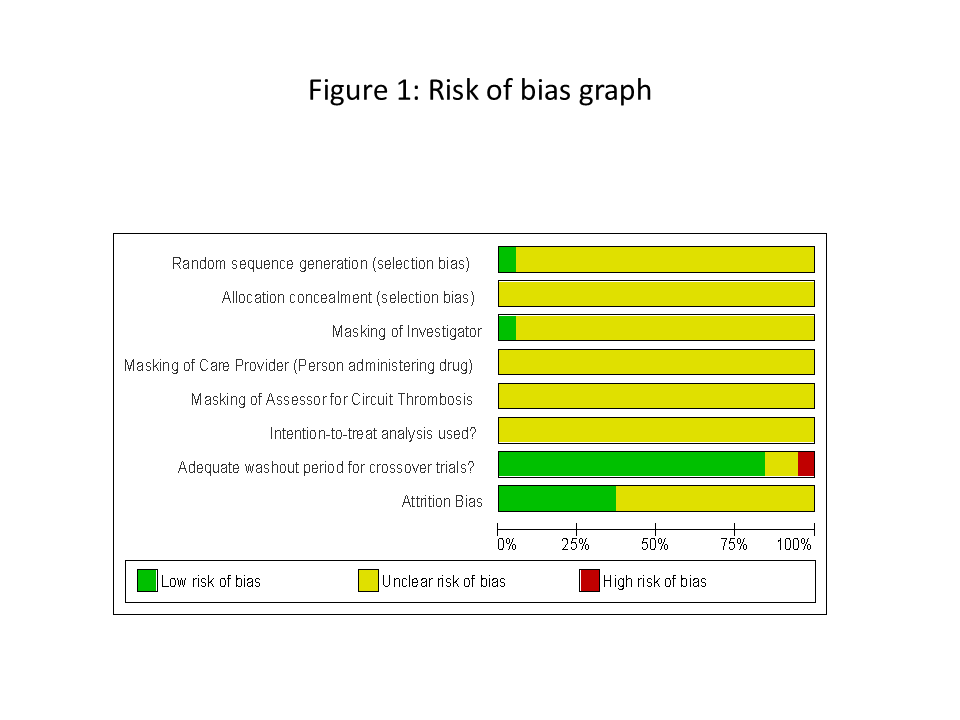

Supplement: Figure S1 — Figure details the risk of Bias assessment followed in the included studies. [file peerj-03-835-s004.png]
