# Supplementary material for: Efficacy and safety of low molecular weight heparin compared to unfractionated heparin for chronic outpatient hemodialysis in end stage renal disease: systematic review and meta-analysis
Source: PeerJ. 2015 Mar 10;3:e835. doi: 10.7717/peerj.835 (PMC4359121; doi:10.7717/peerj.835)
Supplement: Table S2 — Details the risk of Bias assessment followed in the included studies. [file peerj-03-835-s006.docx]

**Supplemental Table 2: risk of bias table**

| Author, year | Random sequence generation | Allocation concealment | Investigator masking | Care provider masking | Assessor masking | Intention to treat analysis | Adequate washout period | Attrition bias | Final assessment of quality |
| --- | --- | --- | --- | --- | --- | --- | --- | --- | --- |
| Aggarwal | ND | ND | ND | ND | ND | ND | Low risk | ND | Poor |
| Borm | ND | ND | ND | ND | ND | ND | Low risk | Low risk | Poor |
| Elisaf | ND | ND | ND | ND | ND | ND | Low risk | ND | Poor |
| Gritters | ND | ND | ND | ND | ND | ND | Low risk | ND | Poor |
| Harrenberg | ND | ND | ND | ND | ND | ND | Low risk | ND | Poor |
| Hottelart | ND | ND | ND | ND | ND | ND | Low risk | ND | Poor |
| Lane | ND | ND | ND | ND | ND | ND | High risk | ND | Poor |
| Lord | ND | ND | ND | ND | ND | ND | Low risk | Low risk | Poor |
| Mahmood | ND | ND | ND | ND | ND | ND | Low risk | Low risk | Poor |
| Naumnik 2003 | ND | ND | ND | ND | ND | ND | Low risk | ND | Poor |
| Naumnik 2007 | ND | ND | ND | ND | ND | ND | Low risk | Low risk | Poor |
| Naumnik 2007 A | ND | ND | ND | ND | ND | ND | Low risk | ND | Poor |
| Naumnik 2009 | ND | ND | ND | ND | ND | ND | Low risk | ND | Poor |
| Naumnik 2009 A | ND | ND | ND | ND | ND | ND | Low risk | ND | Poor |
| Poyrazoglu | ND | ND | Low risk | ND | ND | ND | ND | ND | Poor |
| Ryan | ND | ND | ND | ND | ND | ND | ND | ND | Poor |
| Saltissi | ND | ND | ND | ND | ND | ND | Low risk | Low risk | Poor |
| Schrader | ND | ND | ND | ND | ND | ND | Low risk | Low risk | Poor |
| Verzan | ND | ND | ND | ND | ND | ND | Low risk | Low risk | Poor |

ND: Not Discussed
