# Supplementary material for: Efficacy and safety of low molecular weight heparin compared to unfractionated heparin for chronic outpatient hemodialysis in end stage renal disease: systematic review and meta-analysis
Source: PeerJ. 2015 Mar 10;3:e835. doi: 10.7717/peerj.835 (PMC4359121; doi:10.7717/peerj.835)
Supplement: Supplementary file 6 [file peerj-03-835-s002.doc]

# *Systematic Reviews and Meta-analysis; Protocol of Ghanshyam Palamaner Subash Shantha*

# Title:

# Efficacy and Safety of Low Molecular Weight Heparin compared to Unfractionated Heparin for Chronic Outpatient Hemodialysis in End Stage Renal Disease (Protocol)

# Protocol information

# Background

Chronic kidney disease (CKD), defined by an estimated glomerular filtration
rate (e-GFR) of < 60 mL/min/1.73 m2 persisting for ≥ 3 months, was estimated
to be prevalent in 25.8 million adults in the United States in the year 2004
[1]. Further, it is estimated that CKD prevalence will increase by 5 million
every decade in the United States [2]. This alarming increase in CKD prevalence
had been due to an associated increase in the prevalence of hypertension, type
2 diabetes mellitus and obesity in the United States [2, 3, 4].
Nearly 35% of the American population is obese [3], 30% hypertensive [5] and
25% diabetic [6]. CKD, obesity, hypertension and diabetes in unison are
estimated to cost the American health care system a sum of $110 billion
annually [5].

## Description of the condition

The disease condition that is the primary focus of our review is end stage
renal disease (ESRD). The natural history of CKD is such that when the damaging
insult to the kidney continues for a long time the kidney function
progressively decreases. Kidney function, estimated by eGFR,
progressively drops and reaches a value of < 15, it is called stage 5 CKD
[6]. When these patients become dialysis dependent then they are said to have
ESRD [6]. So the important difference between patients who have stage 5 CKD and
ESRD is that the latter group of patients are dialysis dependent for survival.
We primarily focus on ESRD patients for our review.

## Description of the intervention

Heparins are naturally occurring substances that have anti-coagulant
properties. The molecular weight of heparins can range between 5000 to 40000
Daltons [7]. Heparins act by accentuating the effect of anti-thrombin III, a
naturally occurring blood enzyme that lyses clots [7]. Low molecular weight
heparins (LMW) are recently identified, widely used, heparin derivatives with a
mean molecular weight of less than 8000 Daltons [7]. Commonly used LMW heparins
are Bemiparin, Certoparin, Dalteparin, Enoxaparin, Nadroparin,
Parnaparin, Reviparin and Tinzaparin [8]. LMW heparin has similar mechanism of action
to conventional heparin. It is much more beneficial to conventional heparin
because of lower incidence of heparin induced thrombocytopenia [8, 9, 10]. One important difference from unfractionated heparin is
that activated partial thromboplastin time (APTT) commonly used in monitoring
adequacy of action of unfractionated heparin cannot be used to monitor the
activity of LMW heparin. Instead, factor Xa levels
are used in montoring the activity of low molecular
weight heparin [10]. Further, it has a renal clearance and hence there are
concerns that it should not be administered to patients with renal
disease. LMW heparin has been widely used in prevention and treatment of
thromboembolic episodes [8].

## How the intervention might work

LMW heparin has similar mechanism of action to conventional unfractionated
heparin. Antithrombin III is a naturally occurring
substance in our blood. It is part of the body's mechanism against clot
formation, the fibrinolytic mechanism [7]. LMW
Heparin acts by accentuating the effects of antithrombin
III and is an inhibitor of factor 10, an enzyme that acts as a pro-coagulant
[8]. Hence, by this dual mechanism, LMW heparin acts better than unfractionated
heparin in lysing clots [8].

## Why it is important to do this review

As mentioned earlier there are concerns that LMW heparin may have decreased
clearance in patients with renal disease as it has predominant renal clearance.
In support of this idea, few observational studies also concurred that LMW
heparin was indeed associated with greater bleeding risk compared to
unfractionated heparin in patients with renal disease [12]. Randomized clinical
trials had either excluded patients with renal disease or through inadequately
powered sub-group analysis, had shown correlation between anti-coagulation
efficacy of LMW heparin and renal clearance suggesting that patients with renal
disease may indeed have increased bleeding risk [13]. A systematic review and
meta-analysis on the same topic was conducted by Lim et al. [14] in 2004 where
they had abstracted data from 17 trials. They concluded that LMW heparin was as
effective and safe as conventional heparin in patients with ESRD receiving
regular hemodialysis [14]. However, as the authors had reported, risk of bias
was high for the studies included in this meta-analysis and they were small
population studies. The reason why this review that we are conducting is
important are:

·        
Many
more well conducted, larger population RCTs have been published in the last 8
years after the review by Lim et al. [14] was published. Hence the effect
estimate that we may observe from our meta-analysis may be more robust because
we will have larger sample size.

·        
Newer
low molecular weight heparin analogues have come into the market in the last 8
years and these have been tested in the recent RCTs. Hence, our review may
include studies involving them.

# Objectives

The objective of this systematic review is to evaluate the efficacy and
safety of LMW heparin compared to unfractionated heparin in patients with end
stage renal disease receiving outpatient, chronic, intermittent hemodialysis.

# Methods

## Criteria for considering studies for this review

### Types of studies

Randomized controlled trial: The reasons for selecting only randomized
controlled trials and not observational studies are that:

·        
There
are enough, well conducted RCTs that have addressed our review question.

·        
RCTs
by virtue of randomization effectively controls for known and unknown confounders.
In our review question, main confounders that we may encounter are those that
are associated with bleeding tendencies. Hence, RCTs by controlling for these
confounders will help us arrive at an unbiased effect estimate.

All types of RCTs namely parallel group, cross over, N - of 1 etc. will be
considered eligible for the review. Only human studies will be included. Only
English articles will be included as no one in our group working on the review
is familiar with any other language. We will only include articles where the
intervention allocation was truly random. Quasi-randomized or any other type of
non-random intervention allocation will be criteria for exclusion of the RCT.
We will include RCTs conducted in any part of the world. We will include any
RCT that has used LMW heparin that has been approved by the FDA. RCTs that have
investigated LMW heparins not currently approved by FDA will not be included in
the review. We will not restrict RCTs based on when the study was conducted.
Any RCT relevant to our topic that can be retrieved using the databases that we
have decided to search, irrespective of when it was conducted, will be
considered eligible. RCTs will be included irrespective of study duration,
sample size, presence or absence of run in period, duration of run in period.
RCTs will be included irrespective of whether ethics approval or trial
registration was mentioned in the manuscript. However, these points will be
considered in the risk of bias assessment.

### Types of participants

We will include only ESRD patients receiving chronic, intermittent,
out-patient hemodialysis. The reasons for selecting this patient population are
the following:

·        
Chronic: dialysis is also a treatment
modality for acute conditions such as acute renal failure, acute poisoning etc.
In this review we will only include patients receiving chronic dialysis for
ESRD. Though we are not defining chronicity by any predefined length of time a
patient had to have received hemodialysis, it is left to the authors of the included
papers to have their own definitions to explain the concept described here.

·        
Intermittent: Patients’ receiving
continuous dialysis and continuous venovenous
hemofiltration are not included as there are not many RCTs conducted in these population of patients that had addressed our question
and also to prevent heterogeneity we decided to exclude these patients.

·        
Outpatient: we are excluding patients
receiving home dialysis and hospitalized patients to maintain homogeneity, and
to prevent confounding due to factors that may increase the risk of bleeding.

·        
Hemodialysis: We are not including
patients receiving peritoneal dialysis as anti-coagulants are generally not
administered to these patients before dialysis.

The diagnosis of ESRD should have been physician (primary care physician or
a nephrologist) made. All adult patients aged > 18 years, all races, both
males and females will be included for the review. We will exclude patients
with hyper-coagulable states.

### Types of interventions

We will be including all studies that have used any analogue of low
molecular weight heparin that is approved for use in the United States by the
FDA (Food and Drug Administration). This includes but not limited to Dalteparin, Enoxaparin, and Tinzaparin.
Studies will not be considered ineligible based on route of administration,
dose, duration of intervention, or frequency of administration. Further, we
will not exclude studies based on when the LNM heparin was administered
relative to the dialysis session (before or after dialysis or the previous day
etc.). We will not exclude studies based on route of administration, dose of
administration, duration, frequency of administration of LMW heparin. However,
we will exclude studies where LMW heparin was administered to patients not for
the indication of anti-coagulation for hemodialysis but for therapy of another
condition such as deep vein thrombosis, pulmonary embolism etc. We will also
exclude articles that have used LMW heparin as lock solution.

The comparison intervention should be conventional unfractionated heparin
only. Since there are a lot of RCTs that have compared LMW heparin with
unfractionated heparin we believe we will have good number of included articles
in our review and also for a meta-analysis. Hence, by consensus we have decided
to exclude all other comparison interventions such as citrate, other analogues
of LMW heparin, direct thrombin inhibitors (example: argotroban),
vitamin K antagonists (warfarin), anti-platelets (aspirin, clopidogrel)
and any other anti-coagulant with any other mechanism of action. We are also
not including passive interventions such as no interventions, placebo, and sham
treatment. However, we will not exclude studies based on dose, route,
frequency, and duration of administration of unfractionated heparin. We will
exclude articles that have used unfractionated heparin as lock solution.

### Types of outcome measures

For including studies into the review we do not exclude articles based on
outcomes. So long as all other criteria mentioned above are satisfied we will
include the studies irrespective of the outcomes addressed in the trials.

#### Primary outcomes: For outcomes abstracted from included trials we plan to predominantly concentrate on clinically relevant outcomes.

1.      Extracorporeal circuit thrombosis
during dialysis session: abstracted as presence or absence (yes/no)

2.      Graft or fistula thrombosis:
abstracted as presence or absence (yes/no)

are the two primary outcomes that will be used for the review. Both are
clinically relevant, studied in many RCTs and denotes low molecular heparin
efficacy. Both will be treated as dichotomous variables for the analysis. The
time point that will be recorded for our review for primary outcome 1 i.e.
extracorporeal circuit thrombosis will during the dialysis sessions because the
primary reason for heparin administration is to prevent circuit thrombosis
during dialysis, for primary outcome 2 i.e. graft or fistula thrombosis the
time point of outcome determination that will be included in our study will be
7 days after study commenced and patients received the interventions. This is
because we expect to remove confounding due to other factors that might play a
role in graft and fistula thrombosis and hence 7 days will be adequate time for
the same.

#### Secondary outcomes

1.      Bleeding complications (i.e.
intra-cranial hemorrhage, hemorrhagic stroke or any clinically recorded
bleeding) - data will be abstracted as number of patients with event.

2.      Deep vein thrombosis (DVT) -
again data will be abstracted as number of patients with event.

3.      Pulmonary embolism (PE)
(number of patients with event)

4.      Vascular compression time
(continuous in seconds, and at points in time)

5.      Lipid Profile: [low density
lipoprotein (LDL), high density lipoprotein (HDL), very low density lipoprotein
(VLDL), Total Cholesterol, LDL/HDL ratio)] - (measured as continuous, and at
points in time)

Are the secondary outcomes that will be evaluated. Bleeding complications
and lipid profile will be abstracted because they denote adverse effects of LMW
heparin. Secondary outcomes 1, 2 and 3 will be noted at time point 7 days after
study commencement and intervention administration. Outcome 4 i.e. vascular
compression time will be noted during dialysis sessions and outcome 5 i.e.
lipid profile will be noted at the end of 1 month after study commencement and
intervention administration. The reason being it takes a minimum of 4 weeks for
lipid profiles to change in any person.

## Search methods for identification of studies

We will be searching 3 databases namely: 1) Pubmed,
2) Embase, 3) Cochrane central.
We will not be using language and human restriction when we search for
citations. However, these exclusions will apply as mentioned above when we
finally include articles for review.

### Electronic searches

Pubmed search: The two main concepts
we will focus for our Pubmed search are: 1) Anticoagulants and 2) dialysis. We
will retrieve all MeSH terms related to these two
concepts and also all possible key words from title, abstracts and all fields
based on clinical knowledge, from articles used in the review by Lim et al., micromedix (looking for various versions of heparin used in
the United States) and from expert opinion from hematology and nephrology
friends. The third step in our search, we will use the highly sensitive
Cochrane search for RCTs. We will then combine steps 1 AND 2 AND 3. From the
final list of citations that we retrieve, we will recheck to see if all at
least the articles that were used by Lim et al. were retrieved by our search
strategy. We expect to see 16 of the 17 articles retrieved by our search. The 1
article that will not retrieved by our search is the one that is not indexed to
Pubmed. Our final combined Pubmed
Search stragety will be as follows:  
 *1) ("randomized controlled trials as topic"[MeSH
Terms] OR ((randomized controlled trial[pt] OR controlled clinical trial[pt] OR randomized[tiab] OR
placebo[tiab] OR "drug therapy"[Subheading]
OR randomly[tiab] OR trial[tiab]
OR groups[tiab]) NOT ("animals"[MeSH Terms] NOT "humans"[MeSH
Terms]))) -* Highly sensitive cochrane search
strategy for RCTs

*AND*

*2) ("Anticoagulants"[Mesh] OR "Anti-coagulants"[All
Fields] OR "Anti-coagulant"[All Fields] OR “anticoagulant”[all
fields] OR “antithrombin”[all fields] OR
"Anticoagulant Agents"[All Fields] OR "Anticoagulant
Agents"[All Fields] OR "Anticoagulant Drugs"[All Fields] OR
"Antithrombins"[Mesh] OR "Antithrombins"[All Fields] OR "Heparin, Low
Molecular Weight"[MESH] OR “Adomiparin”[all
fields] OR “antixarin”[all fields] OR “ardeparin”[all
fields] OR “bemiparin”[all fields] OR “certoparin”[all fields] OR “cy 222”[all fields] OR “danaparoid”[all fields] OR “deligoparin”[all
fields] OR “embolex”[all fields] OR “idrabiotaparinux”[all fields] OR “idraparinux”[all
fields] OR “livaparin”[all fields] OR “minolteparin”[all fields] OR “monoembolex”[all
fields] OR “parneparin”[all fields] OR “rd 11885”[all fields] OR “reviparin”[all
fields] OR “semuloparin”[all fields] OR “tafoxiparin”[all fields] OR “tedelparin”[all
fields] OR “logiparin”[all fields] OR “eurodal”[all fields] OR “boxol”[all
fields] OR “low liquemine”[all fields] OR “enoparin”[all fields] OR “decipar”[all
fields] OR “henoxil”[all fields] OR “hepaclex”[all fields] OR “lomoh”[all
fields] OR “nu-mox”[all fields] OR “plaucina”[all fields] OR "Dalteparin"[MESH]
OR "Enoxaparin"[MESH] OR "Heparin, Low Molecular
Weight"[all fields] OR "LMWH"[all fields] OR "Low Molecular
Weight Heparin"[all fields] OR "Low-Molecular Weight
Heparin"[all fields] OR "Low-Molecular-Weight Heparin"[all
fields] OR "Low Molecular-Weight Heparin"[all fields] OR "LMW
Heparin"[all fields] OR "LMW-Heparin"[all fields] OR "Tinzaparin"[all fields] OR "Innohep"[all
fields] OR "tinzaparin sodium"[All Fields]
OR "innohep"[All Fields] OR "logiparin"[All Fields] OR "Fondaparinux"[all
fields] OR "Arixtra"[all fields] OR "Dalteparin"[all fields] OR "Fragmin"[all
fields] OR "Dalteparin Sodium"[all fields]
OR "Tedelparin"[all fields] OR
"Kabi-2165"[all fields] OR “kabi2165”[all fields] OR “kabi 2165”[all fields] OR "Kabi
2165"[all fields] OR "Fragmin"[all fields]
OR "Fragmine"[all fields] OR "Dalteparin Sodium"[all fields] OR "Sodium, Dalteparin"[all fields] OR "FR-860"[all
fields] OR "FR 860"[all fields] OR “FR860”[all fields] OR
"Enoxaparin"[all fields] OR "Lovenox"[all
fields] OR "Enoxaparin"[all fields] OR "Enoxaparine"[all
fields] OR "PK-10,169"[all fields] OR "PK 10,169"[all
fields] OR "PK-10169"[all fields] OR "PK 10169"[all fields]
OR "PK10169"[all fields] OR "EMT-967"[all fields] OR
"EMT 967"[all fields] OR "EMT-966"[all fields] OR "EMT
966"[all fields] OR “Clexan”[all fields] OR
"Clexane"[all fields] OR "Cutenox"[all fields] OR "Dripanina"[all
fields] OR "Enoxaparin Sodium"[all fields] OR "Klexane"[all fields]):* Search Strategy for
anti-coagulants and heparin.

*AND*

*3) ("Renal Dialyses"[all fields] OR "Renal Dialysis"[MeSH] OR "hemodialysis"[all fields] OR "Hemodialyses"[all fields] OR "Extracorporeal
Dialyses"[all fields] OR "Haemodialysis"[all
fields] OR "Dialysis"[MeSH] OR
"Dialysis"[all fields] OR "Microdialysis"[MeSH] OR "Microdialysis"[all
fields] OR "Hemodiafiltration"[mesh] OR
"hemodiafiltration"[all fields] OR "hemo-dialysis"[all fields] OR "hemo-dialyses"[all fields] OR "haemodialyses"[All Fields] OR “renal replacement
therapy”[all fields] OR "RRT"[all fields]):* Search strategy
for dialysis.

**Final Search: #1 AND #2 AND # 3**

 

Cochrane CENTRAL Search: Our Cochrane search strategy will be as
follows:

#1 MeSH descriptor Anticoagulants explode all trees
      
  
#2 MeSH descriptor Antithrombins
explode all trees  
  
#3 MeSH descriptor Heparin, Low-Molecular-Weight
explode all trees  
  
#4 (anticoagulant agent) OR heparin OR (low molecular weight heparin) OR Adomiparin OR (antixarin) OR
(ardeparin) OR (bemiparin) OR (certoparin)
OR (cy 222) OR (dalteparin) OR (danaparoid)
OR (deligoparin) OR (embolex)
OR (enoxaparin) OR (fondaparinux) OR (idrabiotaparinux) OR (idraparinux)
OR (livaraparin calcium) OR (minolteparin)
OR (monoembolex) OR (nadroparin)
OR (parnaparin) OR (rd
11885) OR (reviparin) OR (semuloparin)
OR (tafoxiparin) OR (tedelparin)
OR (tinzaparin) OR (heparin derivative)/exp OR (anticoagulant agent)/exp OR (Anticoagulants) OR
(Anti-coagulants) OR (Anti-coagulant) OR (Anticoagulant) OR (Antithrombins) OR (Low Molecular Weight Heparin) OR (Low
Molecular Weight Heparins) OR (LMWH) OR (Low-Molecular Weight Heparin) OR
(Low-Molecular Weight Heparins) OR (Low Molecular-Weight Heparin) OR (Low
Molecular-Weight Heparins) OR (LMW Heparin) OR (LMW-Heparin) OR (tinzaparin) OR (innohep) OR (logiparin) OR (Arixtra) OR (Fragmin) OR (Tedelparin) OR
(Kabi-2165) OR (Kabi 2165) OR (Kabi2165) OR (Fragmine) OR (FR-860) OR (FR 860) OR (FR860) OR (Eurodal) OR (Boxol) OR (Low Liquemine) OR (Lovenox ) OR (Enoxaparine) OR (PK-10,169)  OR (PK 10,169) OR
(PK10,169) OR (PK-10169) OR (PK 10169) OR (PK10169) OR (EMT-967) OR (EMT 967) OR
(EMT967) OR (EMT-966) OR (EMT 966) OR (EMT966) OR (Clexa)
OR (Clexane) OR (Cutenox)
OR (Decipar) OR (Dripanina)
OR (Enoparin) OR (Flenox)
OR (Henoxil) OR (Hepaclex)
OR (Klexane) OR (Lomoh) OR
(Nu-Rox) OR (Plaucina) OR (Trombenox)  
  
#5 MeSH descriptor Dialysis explode all trees  
  
#6 MeSH descriptor Hemodiafiltration
explode all trees

#7 MeSH descriptor Hemodialysis, Home explode all
trees  
  
#8 (Renal Dialyses) OR Hemodialysis OR Hemodialyses
OR (Extracorporeal Dialyses) OR Dialysis OR Microdialysis
OR Hemodiafiltration OR hemodiafiltration
OR haemodialyses OR dialyses OR haemodialysis
OR (renal replacement therapy)  
  
**#9 ((#1 OR #2 OR #3 OR #4) AND (#5 OR #6 OR #7 OR #8)): will
be the final combined search strategy for Cochrane central.**

 

EMBASE Search:  
  
Here again we are using the two concepts namely: 1) anti-coagulants and 2)
dialysis. We will retrieve all EMTREE terms and key words associated with these
concepts. We will further use commercial drug names from micromedix
and expert opinion from hematologists and nephrologists to retrieve more
keywords. Also, we will use the trials mentioned in the review by Lim et al. to
retrieve more keywords.   
  
#1 Anti-coagulant part -   
‘anticoagulant agent’/exp OR ‘heparin’/exp OR ‘low molecular weight heparin’/exp
OR ‘Adomiparin’ OR ‘antixarin’
OR ‘ardeparin’ OR ‘bemiparin’ OR ‘certoparin’
OR ‘cy 222’ OR ‘dalteparin’ OR ‘danaparoid’
OR ‘deligoparin’ OR ‘embolex’
OR ‘enoxaparin’ OR ‘fondaparinux’ OR ‘idrabiotaparinux’ OR ‘idraparinux’
OR ‘livaraparin calcium’ OR ‘minolteparin’
OR ‘monoembolex’ OR ‘nadroparin’
OR ‘parnaparin’ OR ‘rd
11885’ OR ‘reviparin’ OR ‘semuloparin’
OR ‘tafoxiparin’ OR ‘tedelparin’
OR ‘tinzaparin’ OR ‘heparin derivative’/exp OR ‘anticoagulant agent’/exp
OR “Anticoagulants” OR “Anti-coagulants” OR “Anti-coagulant” OR “Anticoagulant”
OR “Antithrombins” OR “Low Molecular Weight Heparin”
OR “Low Molecular Weight Heparins” OR “LMWH” OR “Low-Molecular Weight Heparin”
OR “Low-Molecular Weight Heparins” OR “Low Molecular-Weight Heparin” OR “Low
Molecular-Weight Heparins” OR “LMW Heparin” OR “LMW-Heparin” OR “tinzaparin” OR “innohep” OR “logiparin” OR “Arixtra” OR “Fragmin” OR “Tedelparin” OR
“Kabi-2165” OR “Kabi 2165” OR “Kabi2165” OR “Fragmine” OR “FR-860” OR “FR 860” OR “FR860” OR “Eurodal” OR “Boxol” OR “Low Liquemine” OR “Lovenox ” OR “Enoxaparine” OR “PK-10,169”  OR “PK 10,169” OR
“PK10,169” OR “PK-10169” OR “PK 10169” OR “PK10169” OR “EMT-967” OR “EMT 967”
OR “EMT967” OR “EMT-966” OR “EMT 966” OR “EMT966” OR “Clexa”
OR “Clexane” OR “Cutenox”
OR “Decipar” OR “Dripanina”
OR “Enoparin” OR “Flenox”
OR “Henoxil” OR “Hepaclex”
OR “Klexane” OR “Lomoh” OR
“Nu-Rox” OR “Plaucina” OR “Trombenox”  
  
#2 Dialysis part -   
‘dialysis’/exp OR dialysis OR ‘hemodialysis’/exp OR hemodialysis OR ‘haemodialysis’/exp OR haemodialysis OR ‘extended
daily dialysis’/exp OR ‘home dialysis’/exp OR ‘renal replacement therapy’/exp
OR ‘renal replacement therapies’/exp OR
‘hemofiltration’/exp OR hemofiltration or ‘hemodiafiltration’/exp OR hemodiafiltration OR ‘hemo-dia-filtration’/exp OR hemo-dia-filtration OR ‘haemofiltration’/exp OR haemofiltration OR haemodiafiltration
OR ‘haemodiafiltration’/exp
OR ‘intermittent dialysis’/exp OR ‘outpatient
dialysis’/exp OR ‘out-patient dialysis’/exp OR ‘outpatient hemodialysis’/exp
OR ‘out-patient hemodialysis’/exp OR ‘outpatient haemodialysis’/exp OR
‘out-patient haemodialysis’/exp
OR ‘outpatient hemofiltration’/exp OR ‘out-patient
hemofiltration’/exp OR ‘out-patient hemodiafiltration’/exp OR
‘outpatient hemodiafiltration’/exp
OR ‘renal dialysis’/exp OR ‘extracorporeal dialysis’/exp OR ‘extra-corporeal dialysis’/exp
OR microdialysis OR ‘microdialysis’/exp OR micro-dialysis OR ‘micro-dialysis’/exp  
  
#3 RCT part -   
We combined (using OR) the ideas of the most sensitive and middle sensitive
approaches from the Wong paper on EMBASE searching strategies for RCTs  
random\*:de,lnk,ab,ti,au OR random:de,lnk,ab,ti,au
OR 'clinical trial':de,lnk,ab,ti,au OR 'clinical
trials':de,lnk,ab,ti,au OR 'health care quality'/exp
OR 'health care quality' OR 'treatment outcome'/exp OR ‘treatment outcome'  
  
                                     **FINAL COMBO  #1 and #2 and #3 = final Embase search.**

### Searching other resources

For this review we will be limited to searching only these above mentioned 3
databases. We will not attempt any grey literature search or hand searching or
searching conference proceedings or contacting individual authors to retrieve
more citations due to time constraints in this course.

## Data collection and analysis

### Selection of studies

Once we retrieve all citations as mentioned above from the three databases
as mentioned above we will import them to EndNote directly from these three
databases. Once we import them into EndNote we will search for duplicates by
title, author, date, volume, and journal. Thus identified duplicates will be
removed and then the final list of citations after removal of duplicates will
be finalized. Then, these final citations will be exported to Microsoft excel
work book. Three reviewer pairs will be created. Reviewer pairs will be created
such that one will be a methodology expert and the other will be a clinical
expert. The total included citations after excluding duplicates will be
randomly divided among the three pairs of reviewers such that each pair gets
equal number of citations to screen. Screening will be
done using Microsoft excel. Now, within each pair, reviewers will independently
screen abstracts and titles for eligibility to retrieve full text articles. At
the stage of abstract and title screening we will screen articles as 'yes' or
'no'. Full texts of all articles marked as 'yes' by both reviewers within a
pair will be retrieved. Articles marked 'no' at this stage by both reviewers
will be excluded. Articles marked 'yes' by one and 'no' by the other will show
up as conflict. Conflicts at this stage will be resolved by consensus.
Citations that conflict even after discussion will be considered as 'yes' and
full text will be retrieved for these articles. Full text retrieval will be
done by re-exporting the 'yes' citations to EndNote. Once full texts of all
'yes' articles from title and abstract screening has been retrieved they will
again be randomly sorted and allotted to three reviewer pairs to determine
eligibility to include into the final review. Within each pair reviewers will
screen full text articles independently and mark them as 'yes' or 'no'.
Articles marked 'yes' by both reviewers at this stage will be included for the
review. Articles marked 'no' by both reviewers will be excluded. Articles
marked 'yes' by one and 'no' by the other will show up as conflict. Conflicts
will be resolved by consensus. If resolution does not happen after discussion
then it will be resolved by discussing as a group with the entire team and
consensus will be reached regarding inclusion into the study.

### Data extraction and management

Included studies will be randomly divided into three groups. Three pairs of
reviewers will be abstracting data and one set of included articles will be
assigned to each pair. Within each pair, both reviewers will independently
abstract data from the included trials and registering the abstracted data into
data abstraction forms which will be created in Google docs. Each reviewer will
be blinded with regards to the entry of the other reviewer. After independent
data abstraction is complete the two reviewers in each pair will compare their
responses. Discrepancies at this stage will be resolved by consensus. Conflicts
at this stage, beyond discussion, will be resolved by group discussion with the
entire team. After confirming completeness and accuracy of data abstraction,
data will be independently entered into software 'Revman
5.0' using double data entry by two reviewers.

Abstracted data will include:  
Methods: type of randomized trial (including: year(s) of conduct, total sample
size, study duration, date study commenced, place or region of study), study
methodology (including eligibility criteria, methods of randomization, type of
randomization sequence followed, allocation sequence concealment, and masking,
washout period), Participant characteristics: total number, setting (hospital
based or free-standing), age, sex, country, race, comorbidities (diabetes,
hypertension, bleeding disorders, autoimmune disorders), frequency of dialysis,
type of dialysis access (graft, AV fistula, catheter [e.g. Davol,
Hickman, Groshong]) Intervention:  Low Molecular
Weight Heparin and unfractionated heparin (dose, name of drug, route of drug,
timing relative to hemodialysis, frequency of administration), Outcomes:
definition of outcome, for all outcomes we will tabulate the number of patients
and number of hemodialysis sessions with an event, if available.  When
binary outcomes are extracted, the number of patients with the event will be
recorded, timing of assessment, method of assessment. For continuous outcomes
mean and standard deviation or standard error mentioned in the manuscript will
be recorded. Results (sample size completed the study, summary data from each
intervention group, loss to follow-up, compliance to intervention, missing participants),
ethical issues (consent, institutional review board approval, registration of
trial).

### Assessment of risk of bias in included studies

The quality of included studies will be assessed by two independent
reviewers. When there is a discrepancy, it will be resolved by consensus.
Conflicts that remain even after discussion will be resolved by group
discussion with the entire team. The studies will be evaluated for the
following criteria:

a) Allocation:

·        
Sequence
generation:
will be termed 'adequate' (low risk) if the random number generation is by
using a computer generated table, random number sequence table, coin tossing or
any other form that is obviously random and 'inadequate' if the sequence
generation has a non-random component (birth data, visit date or day to the
clinic or alphabetical order or self selection). If the method of randomization
is not specified then it will still be termed "inadequate" (high
risk).

·        
Concealment: Will be termed 'adequate'
(low risk) if concealment was due to a central allocation, opaque envelopes etc
and 'inadequate' (high risk) if any other method where there is a possibility
that the investigator or the participant may know in advance or during
allocation the allocation sequence.

b) Masking of investigators and participants with regards to the
intervention: will be termed 'adequate' (low risk) if both investigator and the
participants were masked and 'inadequate' (high risk) if either or both were
unmasked with regards to the intervention identity.

c) Masking of outcome assessment: The study investigators did not obviously
control when the outcome was determined eg: review of
case records. Then it is considered ‘low risk’. Otherwise, considered ‘high
risk’.

d) Incomplete outcome data: the reasons for incompleteness of the outcome
data is explained then ‘low risk’. Not explained ‘high risk’

e) Selective reporting: When methods section of the manuscript details other
assessment scales only one is reported that is significant (high risk). All
scales are reported (low risk).

f) Loss to follow-up and intention to treat analysis: we will assess studies
to see the reasons for loss to follow-up and if they have adequately reported
all losses to follow-up and if loss to follow-up was random and not due to a
specific reason. Intention to treat analysis is said to have been conducted if
participants lost to follow-up were still included in the group they were
randomized at the study beginning, when data results were analyzed.

All the components will be assessed before deciding the study quality. We
will not follow any scoring system to assess quality of the included studies
but will determine quality based on the subjective assessment of the reviewers
from the three subheadings discussed above. We will have a risk of bias form
created in google docs where 2 independent reviewers will assess and record
their assessment of the study quality.

### Measures of treatment effect

We will report relative risks and 95% confidence intervals of the relative
risks for all dichotomous outcomes. The reason is that we are using only RCTs
and hence relative risks will be mentioned in the RCTs and they will be free of
bias due to confounding. For continuous outcomes such as lipid profile levels
and compression times we will calculate means and standard deviations as they
are commonly used summary statistics for continuous variables.

### Unit of analysis issues

The unit of analysis will be individual participants.

### Assessment of heterogeneity

Clinical heterogeneity will be determined based on clinical knowledge as
assessed by the clinical experts in the team based on patient characteristics,
intervention characteristics, co-morbid conditions etc.

Methodological heterogeneity: will be assessed by methodology experts in the
team based on run in period, duration of study, adequacy of randomization etc.

Statistical heterogeneity: we plan to use Q, I^2^ and *τ*^2^
to assess statistical heterogeneity. Q statistics with level of significance less
than 0.1 and the degree of overlap between confidence intervals. I^2^
of 25%, 50% and 75% will be considered low, moderate and high heterogeneity
respectively. We will assess all three components to decide statistical
heterogeneity.

All team members will meet to discuss all these points and by consensus
decide about the heterogeneity of included studies.

### Data synthesis

Studies will be pooled with the random effects model as we expect
significant heterogeneity based on clinical variability and methodological variability
in the studies that become eligible and will be included in the analysis.
However, we will make this decision only after final heterogeneity assessment
and if we believe there is not much heterogeneity then we may consider fixed
effects model.

### Subgroup analysis

### We plan to do a sub-group analysis based on different types of LMW heparins. There are reports which say that LMW heparins may differ with one another with regards to efficacy and safety. Hence, doing this sub-group analysis will help us identify these differences between the types of LMW heparins.

### Sensitivity analysis

# We will decide on sensitivity analysis only after final assessment of quality of studies included finally for the review. If some studies are assessed to have high risk of bias we may perform a sensitivity analysis only using those studies having a low risk of bias.

# Acknowledgements

We thank Ms. Peggy Gross and Ms. Claire Twose, the
librarians, for their help in designing our search strategy for the systematic
review.

# Declarations of interest

No conflict of interest declared.

**Sources of support:** no funding of any form
was provided for the review

**Reference:**

1)     
Snyder JJ,
Foley RN,
Collins AJ.
Prevalence of CKD in the United States: a sensitivity analysis using the
National Health and Nutrition Examination Survey (NHANES) 1999-2004. Am J Kidney
Dis. 2009; 53(2):218-28.

2)     
Rao MV,
Qiu Y, Wang C,
Bakris G. Hypertension and CKD: Kidney Early
Evaluation Program (KEEP) and National Health and Nutrition Examination Survey
(NHANES), 1999-2004. Am J Kidney
Dis. 2008; 51(4 Suppl 2):S30-7.

3)     
Flegal KM, Carroll MD,
Ogden CL, Curtin LR. Prevalence and trends in obesity among US adults,
1999-2008. JAMA. 2010; 303:235-241.

4)     
Finkelstein
EA, Fiebelkorn IC, Wang G. National medical spending
attributable to overweight and obesity: how much, and who’s paying? Health Aff (Millwood) 2003: Suppl Web
Exclusives:W3-219–W3-226.

5)     
Mokdad AH, Ford ES,
Bowman BA, Dietz WH, Vinicor F, Bales VS, et al.
Prevalence of obesity, diabetes, and obesity- related
health risk factors, 2001. JAMA. 2003;289(1):76-9.

6)      KDOQI;
National
Kidney Foundation. KDOQI Clinical Practice Guidelines and Clinical
Practice Recommendations for Anemia in Chronic Kidney Disease. Am J Kidney
Dis. 2006;47(5 Suppl
3):S11-145.

7)      Linhardt,
R.J. Gunay, N. S. (1999). "Production and
chemical processing of low molecular weight heparins". Sem. Thromb. Hem. 25 (3): 5–16.

8)      Gould MK, Dembitzer AD, Doyle RL, Hastie TJ, Garber AM: Low-molecular-weight heparins compared
with unfractionated heparin for treatment of acute deep venous thrombosis. A
meta-analysis of randomized, controlled trials. Ann Intern Med 130: 800–809,
1999.

9)      Gray E, Mulloy B, Barrowcliffe
TW. Heparin and low-molecular-weight-heparin. Thromb Haemost 2008; 99: 807–818.

10)  Nicolaides AN. et al. Prevention and treatment of venous thromboembolism.
International Consensus Statement (ICS). Guidelines according to scientific
evidence. Int Angiol. 2006;25:101-61.

11)  European Pharmacopedia Commission
(1991). Pharmeuropa 3: 161–165.

12)  Gerlach AT,
Pickworth KK, Seth SK:
Enoxaparin and bleeding complications: A review in patients with and without
renal insufficiency. Pharmacotherapy 20: 771–775, 2000

13)  Spinler SA,
Inverso SM, Cohen M, Goodman SG, Stringer KA, Antimann EM: Safety and efficacy of unfractionated heparin
versus enoxaparin in patients who are obese and patients with severe renal
impairment: Analysis from the ESSENCE and TIMI 11B studies. Am Heart J 146:
33–41, 2003.

14)  Lim W,
Cook DJ,
Crowther MA. Safety and efficacy of low molecular
weight heparins for hemodialysis in patients with end-stage renal failure: a
meta-analysis of randomized trials. J Am Soc Nephrol. 2004;15(12):3192-206.
